# Image data in need of a home

**DOI:** 10.15252/msb.20156719

**Published:** 2015-12-28

**Authors:** Thomas Lemberger

**Affiliations:** ^1^ EMBOHeidelbergGermany

## Abstract

Recognized public community databases for image data deposition have been lacking so far. New databases are emerging that provide a promising infrastructure for hosting and distributing high content imaging datasets.

Imaging is becoming a method of key importance for systems biologists: it bridges scales of biological organization and provides insights into how individual biological components organize to collectively perform higher order functions. Advances in resolution and throughput have indeed rendered image‐based methods suitable for quantitative and systematical analyses across multiple scales. For example, cryo‐electron microscopy analyzes the structural organization of ensembles of macromolecular complexes at‐near atomic scale. On the other hand, high‐content imaging screens, which systematically map genetic and environmental perturbations to phenotypes, link the molecular and cellular scales. Furthermore, single‐cell level analyses have uncovered an unforeseen degree of cell‐to‐cell heterogeneity, which has important implications for understanding the behavior of whole cell populations. In addition to the advances in image acquisition, continuous progress in computational methods for image analysis is further revolutionizing the field. Automated image‐based object (e.g., protein complexes or single‐cell organisms) classification and correlation with parallel molecular profiling data of complex biological samples opens exciting avenues for integrating structural and molecular data. At the whole‐organism level, spatially and temporally resolved *in vivo* imaging of entire organs makes it possible to quantitatively characterize anatomical, developmental, and physiological phenotypes and to associate genetic variants to organism‐level traits.

Due to their high information content, the potential of images for data reuse and re‐analysis is considerable. Paradoxically, in spite of their value to the community, it has remained a challenging task to make imaging datasets publically available, mainly due to the lack of appropriate databases. In contrast, most other data types produced by large‐scale analytical platforms (microarrays, sequencing, mass spectrometry, physical interaction assays, structure data, flow cytometry) can be deposited in dedicated repositories. Accordingly, EMBO Press journals, including *Molecular Systems Biology*, request deposition of such data in these repositories as a condition for publication (see http://msb.embopress.org/authorguide#datadeposition). The application of such policies to imaging datasets has, however, been difficult in the absence of recognized repositories. As a consequence, a patchwork of heterogeneous solutions has been adopted. In some cases, authors have developed web resources hosted by their own institution (e.g., http://www.cellmorph.org, for Fuchs *et al*, 2010, or http://www.signalingsystems.ucla.edu/code/max/ for Shokhirev *et al*, 2015). In other cases, datasets were deposited as “flat files” in a general repository for unstructured data, such as Dryad (see e.g., http://dx.doi.org/10.5061/dryad.r4n35 for Schmidt‐Glenewinkel & Barkai, 2014) or in journal‐specific resources such as the *Journal of Cell Biology*'s DataViewer (http://jcb-dataviewer.rupress.org/?view=hcs). While these solutions are certainly better than not providing any access to the data, they all suffer from different drawbacks either in terms of the scalability and long‐term stability of the resources or with regard to the standardization of the data and the services provided to users. From the point of view of the journals, several criteria are important when considering public data repositories:

- The ability of the resource to guarantee long‐term preservation of the data.
- The usability of the data and the value added in terms of curation, searchability, and services provided to authors and end‐users.
- The user‐friendliness of the data deposition process.
- The ability to provide stable and resolvable identifiers.

The size of large‐scale microscopy datasets, which often comprise hundreds of thousands of files amounting to terabytes of data, undoubtedly represents a particular challenge. In addition, it is no trivial task to host and disseminate image data in formats that render them reusable in a variety of cases. For example, distributing the data not only as raw images but also in a processed form, that is, as extracted features, can be extremely useful, since it can facilitate broader use of the data, also by scientists who do not have the resources to reanalyze images from scratch.

To address these issues and to fill this wide gap in the landscape of data repositories, new resources are currently being established. A first example is the new EMBL‐EBI BioStudies database (http://www.ebi.ac.uk/biostudies/), which will link a particular “study”, for example, a published paper, to its associated datasets (McEntyre *et al*, 2015). To some extent, a record in BioStudies is reminiscent of what a structured “data citation” section could provide within a published paper: an aggregated list of resolvable links to the datasets underlying the analyses presented in the paper. An important feature of the database in the context of imaging data is that BioStudies will also be able to host large datasets for data types for which no other public repository exists. Therefore, BioStudies represents an attractive and flexible “backbone” infrastructure that has the potential to be adapted to accommodate various data models from various domains of the life sciences.

A second emerging resource is the Image Data Repository (IDR) led by Jason Swedlow at the University of Dundee (http://idr-demo.openmicroscopy.org). Building upon the OMERO framework for open microscopy data management (Allan *et al*, 2012), IDR is designed to host and distribute results from high‐content screens and other massive imaging datasets in a user‐friendly way that integrates data, metadata, and extracted features.

In view of the importance of the issues related to the accessibility of imaging data, and in order to continue its tradition in pioneering data transparency (Lemberger, 2010), *Molecular Systems Biology* has piloted the deposition of the high‐content imaging dataset from Breinig *et al* (2015) to the two resources mentioned above: The BioStudies record S‐BSMS‐PGPC1 (http://wwwdev.ebi.ac.uk/biostudies/studies/S-BSMS-PGPC1) provides links to the raw data files, and the entry in the IDR database (http://dx.doi.org/10.17867/10000101) makes it possible to view and explore the structured data in a user‐friendly way. Finally, the Bioconductor PGPC software package (http://bioconductor.org/packages/PGPC) provides an executable document with all the code that was used to analyze the data, plus useful intermediate data types, such as the numeric features that were extracted from the images. This example illustrates how such resources can work in concert to provide complementary services. Accumulating further examples will hopefully help the related resources to gather information on the most frequent usage patterns for this type of large datasets. For example, will users access only a few individual images related to their gene of interest? If that would be the case, how can 300,000 image files be optimally structured so that scientists are able to download sub‐sections of a large‐scale screen, that is, related to a particular biological process? Which mechanisms should be used to give access to the entire datasets to researchers who need the data to train new machine learning algorithms? Working on these aspects will ultimately lead to the optimization of the databases and data deposition workflows.

To conclude, it is important to note that the issues discussed above are not strictly limited to the imaging field. As shown in a recent survey conducted by the Infrastructure Systems Biology Europe project (ISBE, Stanford *et al*, 2015), a research infrastructure that provides tools to curate, store, disseminate, and cross‐link different data types and models remains to be developed and will be vital for the meaningful exploitation and integration of biological data. In this context, journals can play an important role as an integral component of the research infrastructure by piloting and implementing policies, workflows, and tools that promote the dissemination of peer‐reviewed data to the scientific community.

## References

[msb156719-bib-0001] Allan C , Burel JM , Moore J , Blackburn C , Linkert M , Loynton S , Macdonald D , Moore WJ , Neves C , Patterson A , Porter M , Tarkowska A , Loranger B , Avondo J , Lagerstedt I , Lianas L , Leo S , Hands K , Hay RT , Patwardhan A *et al* (2012) OMERO: flexible, model‐driven data management for experimental biology. Nat Methods 9: 245–253 22373911 10.1038/nmeth.1896 PMC3437820

[msb156719-bib-0002] Breinig M , Klein FA , Huber W , Boutros M (2015) A chemical‐genetic interaction map of small molecules using high‐throughput imaging in cancer cells. Mol Syst Biol 11: 846 26700849 10.15252/msb.20156400 PMC4704494

[msb156719-bib-0003] Fuchs F , Pau G , Kranz D , Sklyar O , Budjan C , Steinbrink S , Horn T , Pedal A , Huber W , Boutros M (2010) Clustering phenotype populations by genome‐wide RNAi and multiparametric imaging. Mol Syst Biol 6: 370 20531400 10.1038/msb.2010.25 PMC2913390

[msb156719-bib-0004] Lemberger T (2010) From bench to website. Mol Syst Biol 6: 410 20823848 10.1038/msb.2010.72 PMC2950087

[msb156719-bib-0005] McEntyre J , Sarkans U , Brazma A (2015) The BioStudies database. Mol Syst Biol 11: 847 26700850 10.15252/msb.20156658 PMC4704487

[msb156719-bib-0006] Schmidt‐Glenewinkel H , Barkai N (2014) Loss of growth homeostasis by genetic decoupling of cell division from biomass growth: implication for size control mechanisms. Mol Syst Biol 10: 769 25538138 10.15252/msb.20145513 PMC4300492

[msb156719-bib-0007] Shokhirev MN , Almaden J , Davis‐Turak J , Birnbaum HA , Russell TM , Vargas JA , Hoffmann A (2015) A multi‐scale approach reveals that NF‐κB cRel enforces a B‐cell decision to divide. Mol Syst Biol 11: 783 25680807 10.15252/msb.20145554 PMC4358656

[msb156719-bib-0008] Stanford NJ , Wolstencroft K , Golebiewski M , Kania R , Juty N , Tomlinson C , Owen S , Butcher S , Hermjakob H , Le Novère N , Mueller W , Snoep J , Goble C (2015) The evolution of standards and data management practices in systems biology. Mol Syst Biol 11: 851 26700851 10.15252/msb.20156053 PMC4704484

